# Predicting the difficulty of pure, strict, epistatic models: metrics for simulated model selection

**DOI:** 10.1186/1756-0381-5-15

**Published:** 2012-09-26

**Authors:** Ryan J Urbanowicz, Jeff Kiralis, Jonathan M Fisher, Jason H Moore

**Affiliations:** 1 Department of Genetics, Institute for Quantitative Biomedical Sciences, Dartmouth Medical School, Lebanon, NH, USA

**Keywords:** EDM, COR, GAMETES, SNP, Model detection, Epistasis, Simulation, Model, Genetics

## Abstract

**Background:**

Algorithms designed to detect complex genetic disease associations are initially evaluated using simulated datasets. Typical evaluations vary constraints that influence the correct detection of underlying models (i.e. number of loci, heritability, and minor allele frequency). Such studies neglect to account for model architecture (i.e. the unique specification and arrangement of penetrance values comprising the genetic model), which alone can influence the detectability of a model. In order to design a simulation study which efficiently takes architecture into account, a reliable metric is needed for model selection.

**Results:**

We evaluate three metrics as predictors of relative model detection difficulty derived from previous works: (1) Penetrance table variance (PTV), (2) customized odds ratio (COR), and (3) our own Ease of Detection Measure (EDM), calculated from the penetrance values and respective genotype frequencies of each simulated genetic model. We evaluate the reliability of these metrics across three very different data search algorithms, each with the capacity to detect epistatic interactions. We find that a model’s EDM and COR are each stronger predictors of model detection success than heritability.

**Conclusions:**

This study formally identifies and evaluates metrics which quantify model detection difficulty. We utilize these metrics to intelligently select models from a population of potential architectures. This allows for an improved simulation study design which accounts for differences in detection difficulty attributed to model architecture. We implement the calculation and utilization of EDM and COR into GAMETES, an algorithm which rapidly and precisely generates pure, strict, *n*-locus epistatic models.

## Background

The complexity of common disease continues to drive the development of new computational strategies for the detection of complex multi-locus associations. In particular, the detection of gene-gene interactions (i.e. epistasis) is a significant challenge driving bioinformatic development [1-4]. The term *epistasis* was coined to describe a genetic “masking” effect, viewed as a multi-locus extension of the dominance phenomenon, where a variant at one locus prevents the variant at another locus from manifesting its effect [5]. More specifically, *statistical epistasis* describes the phenomenon as it would be observed in genetic association studies. Statistical epistasis is traditionally defined as a deviation from additivity in a mathematical model summarizing the relationship between multi-locus genotypes and phenotypic variation in a population [6]. In order to evaluate an algorithm’s ability to detect epistasis, simulation studies are employed. Simulation studies typically involve the generation of a disease model (i.e. a penetrance function) that is subsequently used to simulate the samples of a dataset. However, the generation of realistic complex disease models constitutes a key challenge.

Over the last decade, several strategies for either the generation, characterization, or classification of complex disease models have been proposed. For example, [7] characterized all fully penetrant two-locus models, where genotype disease probabilities were restricted to zero and one. [8] later expanded this characterization to include models with continuous penetrance values. Additionally, they generated a population of random two-locus models within which they characterized “shape-based” classes of epistatic models. They observed that the shape of a model (1) reveals information about the type of gene interaction present, and (2) impacts the ability to detect the underlying epistasis. The term *shape* is used as a generalized conceptualization of model architecture where *architecture* references the unique composition of a model (i.e. the penetrance values and arrangement of those values across genotypes).

In order to generate complex models of a higher order, [9,10] recruited an evolutionary algorithm (EA) and successfully evolved 2 to 5-locus epistatic models. Over successive generations, models were evolved towards a state of pure epistasis and maximal penetrance table variance (PTV) in an effort to generate interesting models. Later, [11,12] added a customized odds ratio (COR) to the multi-objective fitness landscape of the EA. The COR was applied to quantify and maximize the “strength” of a given model. Both PTV and COR are calculated directly from a penetrance table and have been applied to the generation of complex genetic models. However, to the best of our knowledge the relationship between either metric and model detection has yet to be empirically investigated.

While relatively successful, EAs are computationally expensive, time consuming, and do not guarantee that the desired model characteristics will be met. To address this we introduced GAMETES, a fast, direct algorithm for the generation of random, biallelic, *n*-locus, pure, strict, epistatic models [13]. *Pure* refers to epistasis between *n* loci that do not display any main effects [3,13-15]. *Strict* refers to epistasis where *n* loci are predictive of phenotype but no proper multi-locus subset of them are (see in Additional file 1: Figure S1). We focus on pure, strict, epistatic models because they constitute the worst-case in terms of detecting disease associations. This makes them an attractive gold standard for simulation studies which consider complex multi-locus effects.

GAMETES allows the exact specification of four model constraints including; number of loci, heritability, minor allele frequencies, and population prevalence. In developing and testing GAMETES, we observed that despite keeping all previously mentioned model constraints constant, an algorithm’s ability to correctly detect these models could vary greatly. This ability is determined by the proportion of datasets within which the correct underlying model was identified. While some variation can be explained by the probabilistic translation of models into randomly seeded datasets, the rest can logically be attributed to subtle differences in model architecture.

In addition to considering PTV and COR as potential metrics of detection difficulty, the present study introduces the Ease of Detection Measure (EDM). Calculation of EDM is derived from SURF [16], a filter algorithm for detecting attributes more likely to be useful in discriminating between two classes. *Detection difficulty* refers to ease with which the predictive loci of a model are identified by a given algorithm.

We evaluate the correlation between these metrics and detection difficulty across three very different search algorithms: an exhaustive combinatorial search algorithm (i.e. MDR), a filter algorithm (i.e. SURF), and a stochastic search algorithm (i.e. UCS). We demonstrate how the combination of the GAMETES model generation strategy with a model difficulty metric allows for the intelligent, automated selection of model architectures for simulation studies. Given that researchers often do not know the architecture of complex disease models as they appear in the real world, our strategy avoids arbitrary assumptions about model architecture and seeks to select models for algorithmic evaluation which broadly explores the model space.

## Methods

In this section, we describe (1) relevant background in genetics and modeling, (2) the GAMETES modeling approach, (3) our Ease of Detection Measure (EDM), (4) calculation of PTV and COR, (5) the incorporation of these metrics into GAMETES and the design of a more comprehensive simulation study, and (6) our methods for evaluating the utility these metrics.

### Genetics and modeling

The term *genotype* has been used to refer both to the allele states of a single nucleotide polymorphism (SNP), as well as the combined allele states of multiple SNPs. To avoid confusion, we refer to the latter as a multi-locus genotype (MLG) whenever necessary. Penetrance functions, also referred to as penetrance tables, represent one approach to modeling the relationship between genetic variation and risk of disease. Penetrance is the probability of disease, given a particular genotype or MLG. SNPs not under selective pressure within a population typically exhibit genotype frequencies that are predicted by the Hardy-Weinberg Law [17]. We assume HWE such that, the allele frequencies for a SNP may be used to calculate it’s genotype frequencies as follows; freq(*AA*) =* p*^2^, freq(*Aa*) = 2*pq*, and freq(*aa*) =* q*^2^, where *p* is the frequency of the major (more common) allele ‘*A*’, *q* is the minor allele frequency (MAF) where ‘*a*’ is the minor allele, and *p* + *q *= 1. Penetrance functions are easily extended to describe *n*-locus interactions between *n* predictive loci using a penetrance function comprised of 3^*n*^ penetrance values corresponding to each of the 3^*n *^MLGs.

Table 1 gives an example of an epistatic model that is both pure and strict. Each of the nine entries in Table 1 corresponds to one of the nine possible MLGs combining SNPs 1 and 2. For instance, subjects that have the MLG *aa-bb* have a 14.7*% *chance of having disease. What makes these penetrance functions purely epistatic is that while the genotypes of SNPs 1 and 2 are together predictive of disease status, neither is individually. A further discussion of what makes models purely and strictly epistatic is given in [13].

**Table 1:** A 2-locus purely epistatic penetrance function

		**SNP 2**			**Marginal**
	**Genotype**	**BB(.25)**	**Bb (.5)**	**bb(.25)**	**Penetrance**
	AA(.36)	.266	.764	.664	.614
SNP 1	Aa (.48)	.928	.398	.733	.614
	aa(.16)	.456	.927	.147	.614
	Marginal	.614	.614	.614	K = .614
	Penetrance				

### GAMETES

While the model difficulty metrics considered in this paper could be applied to any penetrance-function-based model, we examine models generated using GAMETES. The GAMETES strategy for generating random, *n*-locus, pure, strict epistatic models is briefly reviewed here. Each *n*-locus model is generated deterministically, based on a set of random parameters, a randomly selected direction, and specified values of heritability, minor allele frequencies, and (optionally) population prevalence. The GAMETES algorithm first (1) generates 2^*n *^random parameters and a random unit vector, then (2) generates a random pre-penetrance table by seeding these parameters using the unit vector, and then (3) uses a scaling function to scale the entries of this random pre-penetrance table to generate a random penetrance table. To obtain a random penetrance table having a specified heritability, or heritability and prevalence, it further (4) scales the entries of this penetrance table to achieve, if possible, these values. If steps (1) or (4) are not successful the algorithm starts over, attempting to generate models until either the desired model population size or the iteration limit is reached. For a detailed explanation of this strategy see [13].

The speed and precision of GAMETES allows the generation of a large population of models with the same genetic constraints, but different architectures. With the incorporation of a model detection difficulty metric, we can intelligently select models from this population.

### The Ease of Detection Measure (EDM)

We describe EDM, a value calculated directly from a penetrance function and it’s respective genotype frequencies, for the purpose of predicting model difficulty. Calculation of the EDM originates in the context of SURF [16], a SNP filtering algorithm for datasets. SURF assigns higher scores to SNPs more likely to be useful in discriminating between healthy and diseased samples. When running SURF on datasets generated by GAMETES, the mean of the score of the SNPs comprising an *n*-locus epistatic model is proportional to the EDM associated with that model. Thus the higher the EDM, the easier it is to distinguish SNPs comprising the model from SNPs generated as noise. EDM is a considerably more accurate measure of this than heritability.

#### Computing population prevalence *K* and heritability *h*^2^

Here we describe how population prevalence and heritability are calculated from a penetrance function and it’s associated genotype frequencies. These calculations offer a useful precursor to understanding the calculation of EDM. In this section we abbreviate the notation for multivariate genotypes to G. The population prevalence for any *n*-locus model can be computed by

(1) $$K=\underset{G}{\sum }P(G)f_{G}\;.$$

Here, the sum is over all 3^*n *^MLGs G in the model, $P\left(G\right)$ is the probability that MLG G occurs, and $f_{G}$ is the penetrance value associated with G given by the penetrance function.

The (broad-sense) heritability of a genetic model is defined as the phenotypic variance due to genotype divided by phenotypic variance in the population. Specifically, using the notation of equation (1), the heritability of any *n*-locus model is

(2) $$h^{2}=\frac{1}{K(1-K)}\underset{G}{\sum }P(G){\left(f_{G}-K\right)}^{2}.$$

#### Computing EDM

Using the notation of equations (1) and (2), the EDM of a penetrance function is defined by

(3) $$\text{EDM}=\frac{1}{2{\left(K(1-K)\right)}^{2}}\underset{G}{\sum }{\left(P(G)\right)}^{2}{\left(f_{G}-K\right)}^{2}.$$

Note the similarities between the calculation of EDM and that of heritability as defined in equation (2). Since the genotype probabilities $\left(G\right)$ are squared in the definition of EDM, lesser-occurring genotypes contribute proportionally less to the EDM score than they do in heritability. The EDM can also be expressed as half the Euclidean distance between two points. Namely

(4) $$\text{EDM}=\frac{1}{2}\underset{G}{\sum }{\left(P(G|s)-P(G|w)\right)}^{2}$$

where $P\left(G|s\right)$ is the probability a random case has genotype G and $P\left(G|w\right)$ is the same for a random control. The equivalence of these two forms of the EDM follows from the first two displayed equations in the appendix of [16].

### Calculating COR and PTV

As described in [12], the COR considered here is the average ratio of odds of disease given an exposure to a high-risk genotype relative to exposure at a low-risk genotype. COR is an adaptation of the traditional odds ratio (OR). The COR is derived directly from a genetic model rather than by comparing cases and controls in a dataset. Additionally in COR, the ratio is achieved by comparing high vs. low risk genotypes, while in OR the ratio is typically between individuals with and without some variant of interest. To calculate COR we begin by calculating the expected proportion of cases (*E*_*case*_) and controls (*E*_*control*_) in a dataset that might be generated from this model for each MLG.

(5) $${\text{E}}_{\text{case}}=\frac{P(G)f_{G}}{K}.$$

(6) $${\text{E}}_{\text{control}}=\frac{P(G)(1-f_{G})}{K}.$$

When the expected proportion of cases equals or exceeds that of controls, a MLG is denoted high risk, otherwise it is low risk. Next, the expected number of high risk cases ‘a’, low risk cases ‘b’, high risk controls ‘c’, and low risk controls ‘d’ are found and used to calculate the odds ratio for the model as follows;

(7) $$\text{COR}=\frac{a*d}{b*c}$$

We consider the relationship between COR and EDM in the Additional file 1 (§2.1).

As described in [10], PTV is simply the variance over all penetrance values $\left(f_{G}\right)$ for all 3^*n*^ genotypes G in the model. Using variance for model selection has the disadvantage of not taking genotype frequencies $P\left(G\right)$ into account.

### GAMETES Incorporation

The simplest application of these metrics involves the prediction of detection difficulty for comparing models of interest. We expand this concept in order to automate the intelligent selection of model architectures of simulation studies. In the context of GAMETES, we can select an encompassing set of pure, strict epistatic models for the evaluation of new algorithms. Specifically, given a set of fixed genetic constraints (i.e. *n*-loci, *h*^2^, MAFs, and K) we begin by generating a population of pure, strict, epistatic models of random architecture using the strategy outlined in [13]. In the present study, models were generated until either a population size of 100,000 was reached, or a maximum of 10,000,000 allotted attempts had been made. The goal here is to maximize the range of difficulty metric values observed in the model population, but prevent the algorithm from running indefinitely when models possessing the specified constraints were rare or non-existent. The likelihood that GAMETES can generate a specific type of model is based on random chance. This may not reflect the probability that such a model might be observed in the real world, as other selective pressures may come into play.

Once one of the aforementioned stopping criteria is met, all models (each having the same *n*-loci, *h*^2^, MAFs, and K) are ordered by their difficulty metric value. At this point we select N models as quantiles from this ordered list, capturing the range of observed metric values. We propose selecting two models from this distribution, representing the highest and lowest metric scores. By selecting the extremes, we minimize the number of models and datasets needed to account for architecture. While this strategy is not guaranteed to find models with the mathematical maximum or minimum metric values for a given set of genetic parameters, it offers a reasonable estimate of these values.

### Evaluating metrics

To examine the correlation between either PTV, COR or EDM, and the ability to detect respective underlying models in simulated datasets, we have run an algorithmic evaluation of three very different data search algorithms. Each search algorithm evaluation was designed to yield varying frequencies of detection success. Without variable detection success, the correlation between detection and respective metrics would be nondescript.

#### Data search algorithms

Multifactor Dimensionality Reduction (MDR), a well documented combinatorial genetics algorithm that exhaustively searches for epistatic interactions [18]. MDR finds models by scanning through all possible combinations of SNPs up to a pre-specified order of interaction, and has the ability to identify pure, strict epistatic models. For all evaluations, MDR was set to search up to one order higher than the order of the simulated model. The best model was selected based on 10-fold cross validation (CV) consistency, and in the event of a CV tie, based on testing accuracy. A model was considered to have been successfully detected if MDR correctly identified the precise underlying model.

Spatially Uniform ReliefF (SURF), discussed in Section The Ease of Detection Measure (EDM) is a filtering and ranking algorithm for the rapid identification of SNPs that are more likely to be useful in discriminating between healthy and diseased samples. A model was considered to have been successfully detected if SURF ranked all SNPs from the correct underlying model in the top 20%. Preliminary analysis indicated that this cutoff was sufficient to observe a range of detection success frequencies within the datasets simulated in this study.

The sUpervised Classifier System (UCS) [19] is a Michigan-style learning classifier system (M-LCS) [20], or more generally, an evolutionary algorithm. This stochastic search algorithm has been successfully applied to the problem of pure, strict epistasis in [21]. This type of algorithm is much more computationally intense, so we select run parameters which are less than optimal, but sufficient to obtain the variable rate of success needed to calculate correlation. The implementation of UCS applied in this study is the same as the one used in [21] adopting mostly default parameters with the exception of 50,000 learning iterations, a population size of 1000, tournament selection, uniform crossover, subsumption, and a *ν* of 1. A model was considered to have been successfully detected if UCS correctly specified all SNPs from the correct underlying model more frequently than all other non-predictive SNPs in the respective dataset. This novel detection estimation strategy, unique to M-LCS algorithms, is detailed further in [22]. Successful detection was achieved if the correct attributes were identified in greater than 50% of the 10-fold CV runs for each dataset.

#### Experimental evaluation

Each search algorithm described above was run on a spectrum of simulated datasets, generated using GAMETES. First, we generated models with different numbers of loci, heritabilities, and MAFs, holding prevalence constant. Specifically, we generated 12 populations of both 2-locus and 3-locus, pure, strict epistatic models in which heritability, MAF, and K were specified. Heritabilities of (0.005, 0.01, 0.025, 0.05, 0.1, or 0.2) and MAFs of (0.2 or 0.4) were used. We have chosen to fix K as a constant such that any difference in detectability is strictly due to architecture, independent of all genetic constraints. K was set to 0.3 for each of these models. We selected this value of K based on the limits described in [13] to ensure that all selected combinations of heritability and MAF would yield models. Two models from each aforementioned model population were then selected to be representative of the EDM range (highest and lowest EDM scores), yielding a total of 24, 2-locus models and 24, 3-locus models from which to generate datasets. For the purposes of this study we pick model architectures with EDM and calculate the COR and PTV of these models separately.

For each model, a spectrum of simulated datasets was generated with balanced sample sizes of either (200,400,800, or 1600) and 20 SNPs (*n* of which were modeled as predictive SNPs, the rest being non-predictive, “noise”). Random dataset replicates were generated in the following manner: (1) Genotypes for SNPs specified in the genetic model are probabilistically generated for each sample in the dataset based on their specified MAFs. (2) Affection status (i.e. case/control status) for each sample is determined probabilistically based on the penetrance value (given by the model’s penetrance function) at that MLG. (3) Genotypes for all other “noise” SNPs not specified in the model are generated probabilistically based on a randomly assigned uniform distribution of MAFs ranging from 0.05 to 0.5. We generated 100 randomly seeded replicates for each combination of model and dataset parameters (2 orders of loci ∗ 6 heritabilities ∗ 2 MAFs ∗ 2 EDMs ∗ 4 sample sizes = 192 combinations) yielding a total of 19,200 simulated datasets independently run on MDR, SURF, and UCS.

In addition to the evaluation described above we performed a secondary evaluation of MDR examining models with K = 0.1 to account for the potential impact of varying K. Again we applied GAMETES, attempting to generate the same model constraint combinations for K = 0.1 as we did for K = 0.3. GAMETES was unable to generate certain higher heritability model (an observed limitation of the software [13]). In total we generated 22, 2-locus models and 16, 3-locus models with which to generate simulated datasets as described above.

Lack of normality in the distribution of detection scores, and the inability to adequately normalize these values led us to employ the Spearman Rank Correlation to determine the correlation between detection and our metrics of interest, as well as to examine the correlation between detection and key constraints (e.g. heritability). We expect the correlation between detection and each considered metric to be limited by the fact that our dataset generation strategy creates random dataset replicates which probabilistically generate datasets based on a model. This means that the genotype frequencies and genotype disease probabilities in the generated datasets may vary slightly from those specified in a respective model.

All statistical evaluations were done using R [23], with a significance threshold of P ≤ 0.05. Model detection frequency was determined by the proportion of the 100 dataset replicates within which the correct underlying model was identified. Detection success was considered to be meaningful when it was greater than 0.8.

## Results and discussion

**Figure 1:**
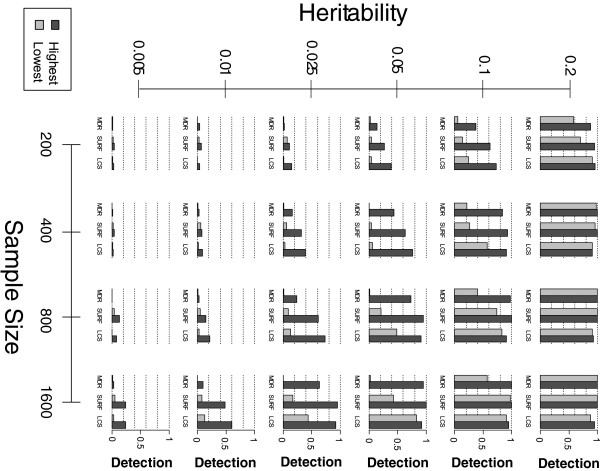
**2-Locus Model Detection: Each bar represents the model detection frequency (averaged between a MAF of 0.**2 and 0.4) for the respective algorithm within 100 simulated datasets. Highest and lowest refers to the respective EDM of a given model within the model population generated by GAMETES. Each sub-plot corresponds to a specific combination of heritability and sample size. A similar figure for 3-locus models is included in the Additional file 1.

Figure 1 summarizes the detection success each data search algorithm displayed in finding the correct underlying 2-locus model over the spectrum of simulated datasets previously described. This figure illustrates the influence which genetic architecture alone can have on the ability to detect a given model. Within each pair of bars, all other model and dataset constraints are equivalent, and we can clearly observe the influence of model architecture on detection. This influence is most obvious when modest detection is achieved (see the diagonal of sub-plots stretching from the top left corner of Figure 1 to the bottom right corner). This figure also illustrates the overall correlation between EDM and detection in 2-locus models. Within each pair of bars, “highest” EDM models consistently yield greater detection than “lowest” EDM models. We average models with MAFs of 0.2 and 0.4 in this figure since we found no correlation between MAF and detection (see Tables 2, 3 and 4).

**Table 2:** **MDR Analysis (K = 0.3) Spearman Rank Correlations: A summary of Spearman rank correlation coefficients (*ρ*) and respective p-values relating detection to the other variables given in the table**

	**2-locus**		**3-locus**	
	***ρ***	**p-val.**	***ρ***	**p-val.**
Heritability	0.7757	**	0.8270	**
Sample Size	0.3508	**	0.3253	**
mAF	0.1257	-	0.075	-
EDM	0.8621	**	0.8564	**
COR	0.8491	**	0.8603	**
PTV	0.1544	-	0.2707	*
EDM vs. COR	0.9722	**	0.9652	**

Each calculation is performed over all 9,600 datasets generated for either 2 or 3-locus models as previously described. Also given is the correlation between EDM and COR over all datasets with K = 0.3. (− Not Sig., * P < 0.05, ** P << 0.001).

**Table 3:** **SURF Analysis (K = 0.3) Spearman Rank Correlations: A summary of Spearman rank correlation coefficients (*ρ*) and respective p-values relating detection to the other variables given in the table**

	**2-locus**		**3-locus**	
	***ρ***	**p-val.**	***ρ***	**p-val.**
Heritability	0.7690	**	-0.0300	-
Sample Size	0.3723	**	0.0241	-
mAF	0.0864	-	-0.0515	-
EDM	0.8798	**	-0.0515	-
COR	0.8602	**	-0.0635	-
PTV	0.1323	-	-0.1403	-

Each calculation is performed over all 9,600 datasets generated for either 2 or 3-locus models as previously described. The correlation between EDM and COR for these datasets are the same as those given in Table 2. (− Not Sig., * P < 0.05, ** P << 0.001).

**Table 4:** **UCS Analysis (K = 0.3) Spearman Rank Correlations: A summary of Spearman rank correlation coefficients (*ρ*) and respective p-values relating detection to the other variables given in the table**

	**2-locus**		**3-locus**	
	***ρ***	**p-val.**	***ρ***	**p-val.**
Heritability	0.7984	**	0.6926	**
Sample Size	0.1567	-	0.0841	-
mAF	-0.0413	-	-0.1410	-
EDM	0.8990	**	0.7512	**
COR	0.8673	**	0.7106	**
PTV	0.1323	-	-0.1403	-

Each calculation is performed over all 9,600 datasets generated for either 2 or 3-locus models as previously described. The correlation between EDM and COR for these datasets are the same as those given in Table 2. (− Not Sig., * P < 0.05, ** P << 0.001).

Tables 2, 3 and 4 summarize the spearman correlation results for MDR, SURF, and UCS respectively. Notice that for each search algorithm, heritability is a significant predictor of detection in both 2 and 3-locus models. This relationship between heritability and detection (while expected) serves as a standard of comparison for the difficulty metrics evaluated here. Also, notice that sample size is significantly correlated with detection in MDR and SURF, but to a much less extent than heritability. While the results in Tables 2, 3 and 4 are relatively self explanatory, notice that the 3-locus results for SURF failed to yield any significant correlations. This is not due to the failure of any metric, rather due to the failure of SURF to correctly detect the SNPs of any 3-locus pure, strict, epistatic model. This finding is worth noting, but beyond the focus of the present study.

Next we turn our attention to the three metrics of interest: EDM, COR and PTV. Tables 2, 3 and 4 indicate that both EDM and COR were strongly and significantly correlated with detection within each of the three search algorithms. Of particular note, both EDM and COR are more strongly correlated with detection than heritability in every evaluation. Alternatively, we find that PTV is not significantly correlated with detection in any of the evaluations. Additionally we note that EDM is slightly more strongly correlated with detection for 2-locus models (we see an even larger difference in the UCS analysis). For 3-locus models COR is slightly more strongly correlated with detection than EDM in the MDR analysis, while the opposite is true for the UCS analysis. At the bottom of Table 2 we include the correlation between EDM and COR within models where K = 0.3. This correlation is relevant to the analyses presented in Tables 2, 3 and 4.

As mentioned in section 1, we performed a secondary evaluation in MDR using models generated with K = 0.1. Table 5 summarizes these correlation results. Here we observe relationships similar to those found with K = 0.3. Specifically, heritability, EDM and COR are all significantly correlated with detection, with EDM and COR being more strongly correlated with detection than heritability. Again we see that EDM is slightly more strongly correlated with detection for 2-locus models, while COR is more strongly correlated for 3-locus models. At the bottom of Table 5 we include the correlation between EDM and COR in these models with K = 0.1. We note that the correlation between EDM and COR is less strong for 3-locus models than for 2-locus models, and less strong for models with K = 0.1 than models with K = 0.3.

**Table 5:** **MDR Analysis (K = 0.1) Spearman Rank Correlations: A summary of Spearman rank correlation coefficients (*ρ*) and respective p-values relating detection to the other variables given in the table**

	**2-locus**		**3-locus**	
	***ρ***	**p-val.**	***ρ***	**p-val.**
Heritability	0.7663	**	0.7722	**
Sample Size	0.3081	*	0.4305	**
mAF	0.1257	-	0.075	-
EDM	0.8237	**	0.7786	**
COR	0.8075	**	0.8241	**
PTV	0.1999	-	-0.072	-
EDM vs. COR	0.9401	**	0.9176	**

ion is performed over all 8,800 datasets successfully generated for 2-locus models or all 6,400 datasets successfully generated for 3-locus models as previously described. Also given are the correlations between EDM and COR over these respective datasets with K = 0.1. (− Not Sig., * P < 0.05, ** P << 0.001).

Together, the findings presented above suggest that either EDM or COR would serve as a suitable metric with which to gauge model difficulty in place of heritability alone. Subjective interpretation of these results suggest that under different circumstances one metric may be more favorable to another. Our data does not support one metric as being universally better than the other, and additional investigation would be required to determine the circumstances within which one would be preferable to the other. As a result we have made both metrics available for model selection in the GAMETES software as both metrics consistently outperform heritability alone. In addition, these results illustrate how model architecture alone can greatly impact detection.

**Figure 2:**
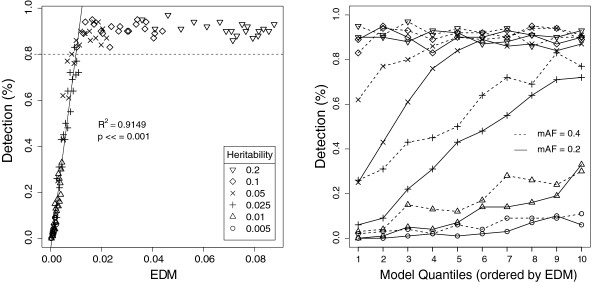
**Plots illustrating the follow-up MDR detection analysis over 10 quantiles.** For all datasets in this analysis, K = 0.3, number of SNPs is 20, and sample size is 800. MAF and heritability vary as before. (Left Panel) The solid regression line gives the best fit for all findings with an observed detection frequency below the significant detection threshold of 0.8 (the dotted line). Similar figures for COR and PTV are given in the Additional file 1. (Right Panel) A different perspective on the data in the left panel. This plot illustrates the capacity of model architecture to impact model detection independent of any genetic or dataset constraints. The x-axis gives the ten models selected to cover the range of EDMs observed. Each line highlights the ability to find these ten models for a respective combination of heritability and MAF.

In order to more clearly observe the relationship between a given difficulty metric and detection, we performed a follow-up MDR analysis using EDM and datasets with sample size held constant. Instead of two models representative of the EDM range, we selected ten in order to observe this relationship at a higher resolution. Entirely new 2-locus models and datasets were generated for this follow up study, using the same values of heritability and MAF (with K = 0.3 as before). Figure 2 (left and right panels) provide different perspectives of this follow up analysis. The left panel illustrates the relationship between detection and raw EDM scores, highlighting their strong positive correlation (*R*^2 ^= 0.9149, *P *<< 0.001) with linear regression, up until the significance threshold of 0.8 is reached. A Box-Cox transformed linear regression (*λ *= 0.57) of this same relationship was found to be similarly strongly correlated (*R*^2 ^= 0.886, *P *<< 0.001). In this example, all models with an EDM above approximately 0.01 are significantly detected by MDR. The right panel of Figure 2 looks at the same set of results, keeping detection on the y-axis, but replacing raw EDM scores with references to quantiles representing models of increasing EDM within the model population. These quantiles are analogous to the previous distinction of highest and lowest EDM, except instead of two models, we choose ten. The right panel illustrates the relationship between detection and EDM within fixed combinations of heritability and MAF, and emphasizes the dramatic impact which model architecture alone can have on an algorithm’s ability to detect it.

## Conclusions

In the present study we formally identify and evaluate metrics which may be used to predict the detection difficulty of a given model. The simplest application of such a metric is to compare models without having to run a search algorithm on datasets generated from them. Using three fundamentally different data search algorithms, we demonstrate that two metrics (EDM and COR) are strongly and significantly correlated with detection (more that heritability alone). We implement both metrics into the GAMETES model/dataset generation software and demonstrate how they may be used to automatically guide the selection of representative model architectures.

Our results clearly illustrate how model architecture alone can dramatically impact detection, which emphasizes the importance of taking model architecture into consideration when designing a simulation study. Without taking architecture into account, researchers run the risk of making incomplete claims about an algorithm’s ability to detect underlying models within datasets having specific characteristics (e.g. heritability, MAF, K, number of attributes, and sample size). By incorporating EDM into the selection of models, researchers can more comprehensively evaluate their algorithms across the space in which real biological associations might appear. Beyond data simulation, the ability to determine a model’s difficulty has the potential to advance our understanding of true biological models by characterizing and comprehending the theoretical space wherein they must lie.

The GAMETES software, incorporating EDM and COR, is open source and freely available for download, and offers an intuitive and flexible framework for the simulation of complex genetic models, and the option to generate simulated datasets from these models. This software offers both a graphical user interface, as well as command line accessibility to facilitate the quick generation of a large simulated dataset archive. The GAMETES software along with a detailed users guide is included as Additional files 2 and 3, respectively.

## Abbreviations

EDM: Ease of Detection Measure; SNP: Single nucleotide polymorphism; EA: Evolutionary Algorithm; COR: Customized Odds Ratio; GAMETES: Genetic Architecture Model Emulator for Testing and Evaluating Software; MLG: Multi-locus genotype; HWE: Hardy-Weinberg Equilibrium; MAF: Minor Allele Frequency; K: Population Prevalence; MDR: Multifactor Dimensionality Reduction; SURF: Spatially Uniform ReliefF; UCS: sUpervised Classifier System.

## Competing interests

The authors declare that they have no competing interests.

## Author’s contributions

RU co-developed the methodology, carried out experiments, and co-wrote the manuscript. JK developed the mathematical proofs, co-developed the methodology, and co-wrote the methods section of the manuscript. JF implemented EDM and COR into the GAMETES software and co-developed the methodology. All authors read and approved the final manuscript.

## Supplementary Material

Additional file 1 **Supplemental Materials.** Includes supplemental background, methods descriptions, and results. Supplemental figures included in this document. Click here for file

Additional file 2 **GAMETES Software Version 1.0 Beta.** The graphical user interface for the GAMETES software. This software is open source and includes a User’s Guide. EDM and COR have been implemented into this version. Click here for file

Additional file 3 **GAMETES User’s Guide.** A reference guide for using the GAMETES software. Click here for file
