# Supplementary material for: Predicting the difficulty of pure, strict, epistatic models: metrics for simulated model selection
Source: BioData Min. 2012 Sep 26;5:15. doi: 10.1186/1756-0381-5-15 (PMC3549792; doi:10.1186/1756-0381-5-15)
Supplement: Additional file 1 — Supplemental Materials. Includes supplemental background, methods descriptions, and results. Supplemental figures included in this document. [file 1756-0381-5-15-S1.pdf]

Predicting the Difficulty of Pure, Strict, Epistatic Models: Metrics for Simulated Model Selection - *Supplementary Materials*

Ryan J. Urbanowicz, Jeff Kiralis,
Jonathan M. Fisher, Jason H. Moore

July 27, 2012

In this document, we provide supplementary materials for the background, methods, and results section.

1 Background

Figure 1 illustrates the difference between *pure* and *impure* epistasis, as well as the difference between *strict* and *nested* epistasis.

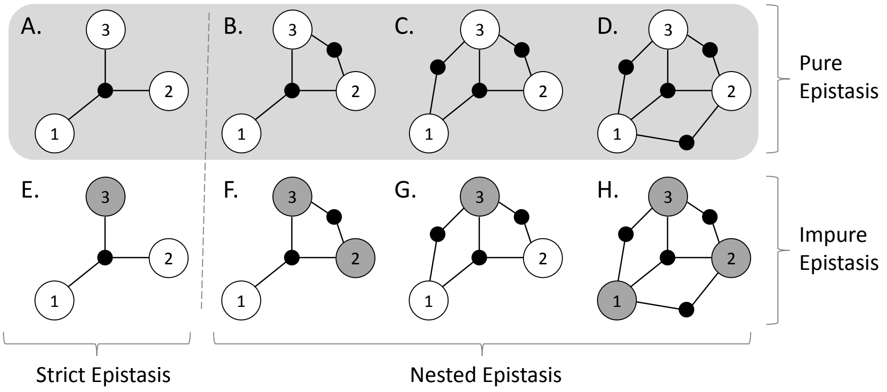

Figure 1: Illustrations (A) – (H) indicate various types of 3-locus epistatic interactions. Each numbered circle represents a SNP (where white circles indicate a SNP with no main effect, and darkened circles represent main effects). Each solid black node, with incident edges connecting respective SNPs, represents a 2 or 3-locus epistatic interaction. Illustrations (A) – (D) exemplify pure epistasis, while (E) – (H) exemplify impure epistasis. Illustrations (A) and (E) exemplify strict epistasis, while (B) – (D) and (F) – (H) exemplify nested epistasis.

2 Methods

2.1 Comparing the calculation of COR with EDM

The COR can be expressed as

$$COR = \frac{P(H|s)}{P(H|w)} \cdot \frac{P(L|w)}{P(L|s)} \tag{1}$$

where $P(H|s)$ is the probability that a random case has a high-risk genotype and $P(H|w)$ is the same for a random control. The probabilities $P(L|s)$ and $P(L|w)$ are defined similarly, but with low-risk in place of high. The EDM can be expressed as

$$\frac{1}{2}\left(\sum_{\text{high-risk } \mathcal{G}} \big(P(\mathcal{G}|s) - P(\mathcal{G}|w)\big)^2 + \sum_{\text{low-risk } \mathcal{G}} \big(P(\mathcal{G}|s) - P(\mathcal{G}|w)\big)^2\right) \tag{2}$$

where the sum in the first term is over all high-risk genotypes and that in the second is over all low-risk ones. Since $\sum_{\text{high-risk } \mathcal{G}} P(\mathcal{G}|s) = P(H|s)$ and $\sum_{\text{high-risk } \mathcal{G}} P(\mathcal{G}|w) = P(H|w)$ some correlation can be seen between the first factor in equation (1) and the first summand in (2), and similarly for the second factor of (1) and the second summand of (2).

3 Results

Figure 2 summarizes the ability each data search algorithm displayed in finding the correct underlying 3-locus model over the spectrum of simulated datasets generated with K = 0.3. This figure illustrates the influence which genetic architecture alone can have on the ability to detect a given model even in higher order models. Within each pair of bars, all other model and dataset constraints are equivalent, and we can clearly observe the influence of model architecture on detection. This influence is most obvious when modest detection is achieved (see the diagonal of sub-plots stretching from the top left corner of Figure 2 to the bottom right corner). This figure also illustrates the overall correlation between EDM and detection in 3-locus models. Within each pair of bars, "highest" EDM models consistently yield greater detection than "lowest" EDM models. We average models with MAFs of 0.2 and 0.4 in this figure since we found no correlation between MAF and detection in this evaluation.

Moving to the data from the follow up MDR evaluation, where 10 models from each constraint combination were selected to span the EDM range, we examine the relationship between COR and detection in Figure 3, and the relationship between PTV and detection in Figure 4. Additionally, Figure 5 shows the relationship between the two metrics determined to be strongly correlated with detection (EDM and COR).

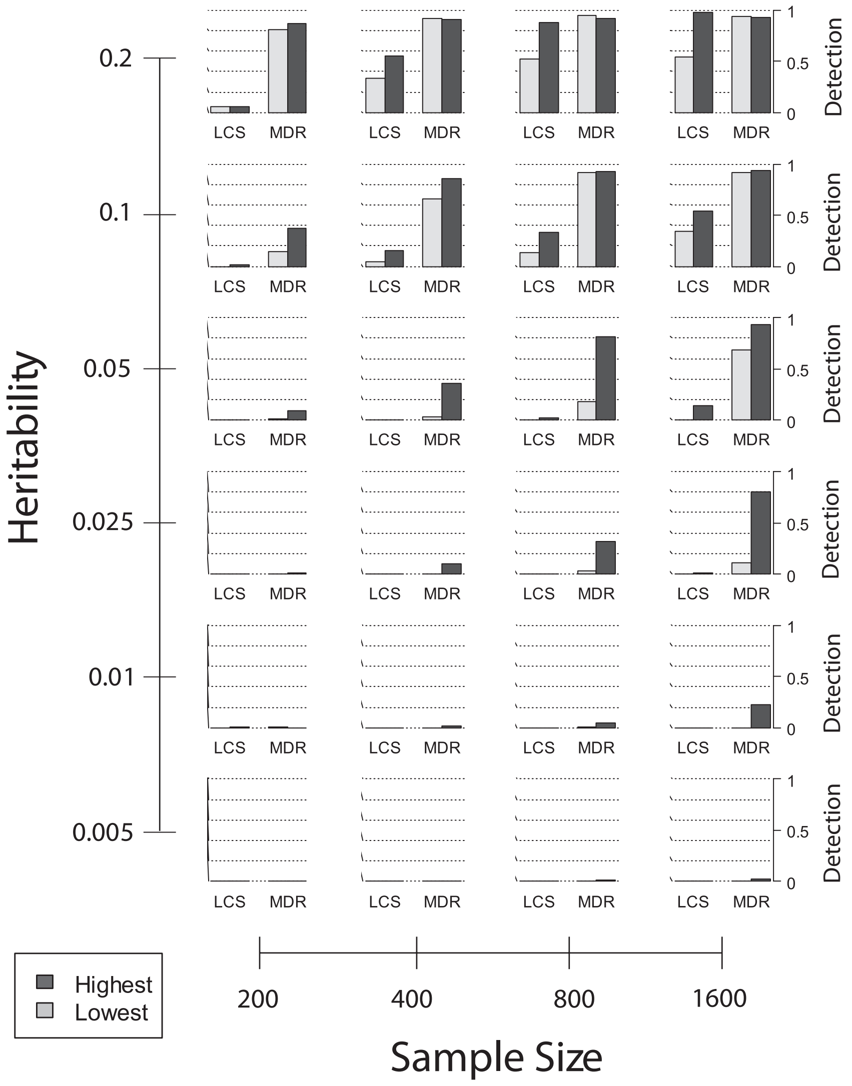

Figure 2: 3-Locus Model Detection: Each bar represents the model detection frequency (averaged between a MAF of 0.2 and 0.4) for the respective algorithm within 100 simulated datasets. Highest and lowest refers to the respective EDM of a given model within the model population generated by GAMETES. Due to the failure of SURF to detect 3-locus models we have left the results for SURF out of this figure since they add nothing. Each sub-plot corresponds to a specific combination of heritability and sample size.

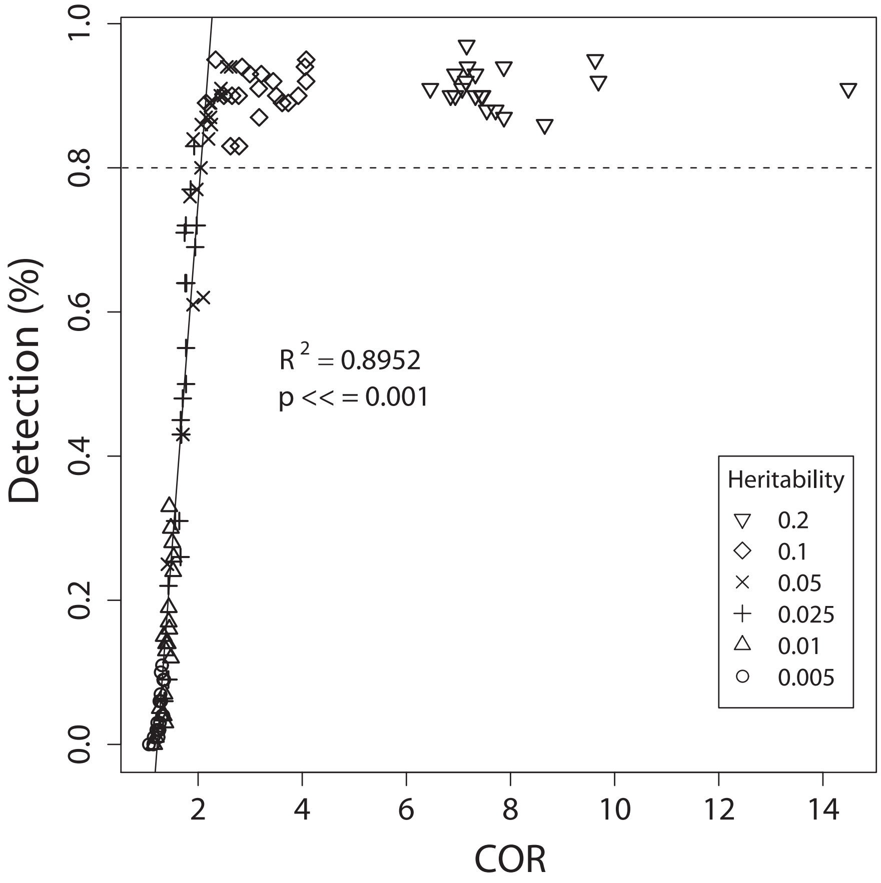

Figure 3: MDR follow-up analysis: Ability of the Customized Odds Ratio to predict detection. All datasets evaluated in this figure have $K = 0.3$, a sample size of 800, 20 SNPs, and were generated from a model with 2-loci. Heritability, and MAF vary as previously described. The solid regression line gives the best fit for all findings with an observed detection below the significant detection threshold of 0.8 (the dotted line). A Box-Cox transformed linear regression ($\lambda = 0.49$) of this same relationship was found to be similarly strongly correlated ($R^2 = 0.9078$, $P << 0.001$).

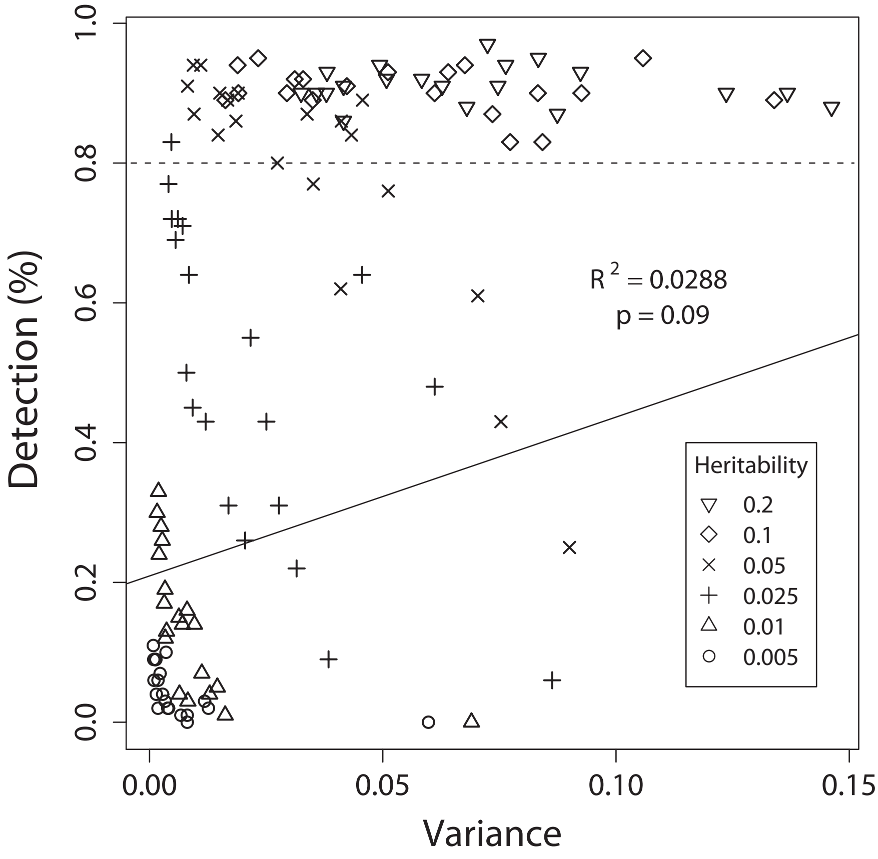

Figure 4: MDR follow-up analysis: Ability of Penetrance Function Variance to predict detection. All datasets evaluated in this figure have $K = 0.3$, a sample size of 800, 20 SNPs, and were generated from a model with 2-loci. Heritability, and MAF vary as previously described. The solid regression line gives the best fit for all findings with an observed detection below the significant detection threshold of 0.8 (the dotted line). A Box-Cox transformed linear regression ($\lambda = 0.31$) of this same relationship was found not to be correlated.

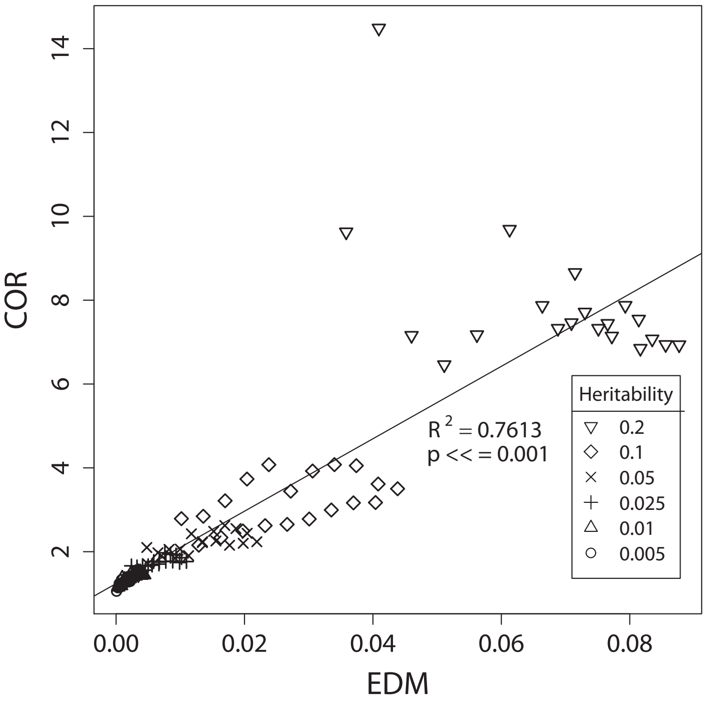

Figure 5: MDR follow-up analysis: EDM vs. Customized Odds Ratio. All datasets evaluated in this figure have $K = 0.3$, a sample size of 800, 20 SNPs, and were generated from a model with 2-loci. Heritability, and MAF vary as previously described.
