# Supplementary material for: Predicting the difficulty of pure, strict, epistatic models: metrics for simulated model selection
Source: BioData Min. 2012 Sep 26;5:15. doi: 10.1186/1756-0381-5-15 (PMC3549792; doi:10.1186/1756-0381-5-15)
Supplement: Additional file 3 — GAMETES User’s Guide. A reference guide for using the GAMETES software. [file 1756-0381-5-15-S3.pdf]

GAMETES User's Guide

Version 0.1.0 Beta

Ryan J Urbanowicz[1] and Geoffrey Kiralis[2] and Jonathan M Fisher[3] and Jason H Moore[4]

January 10, 2012

[1]ryan.j.urbanowicz@dartmouth.edu - Algorithm and Software Development
[2]geoffrey.kiralis@dartmouth.edu - Algorithm Development
[3]jonathan.fisher@dartmouth.edu - Software Development
[4]jason.h.moore@dartmouth.edu - Principle Investigator

Contents

1 Introduction **2**
1.1 What is GAMETES? 2
1.2 Algorithm Overview 2
1.3 Further Reading 2
1.4 Obtaining the Software 3
1.5 Minimum System Requirements 3
1.6 Starting the Program 3

2 Using GAMETES **4**
2.1 GAMETES GUI Overview 4
2.2 Model Generation 4
2.2.1 Number of attributes 4
2.2.2 Heritability 5
2.2.3 Population Prevalence 6
2.2.4 Minor Allele Frequency 6
2.2.5 Other Parameters 6
2.2.6 Saving Models 7
2.3 Dataset Generation 8
2.3.1 Randomly Generate Non-Predictive Attributes 8
2.3.2 Load Non-Predictive Attributes 8
2.4 EDM and COR 9
2.5 Model Output Files 10
2.6 Limits of GAMETES 10
2.7 Command-Line Operation 11

3 Intended GAMETES Expansions **13**
3.1 Heterogeneous Models 13
3.2 Impure and Nested Epistasis 13
3.3 Custom Genetic Models 13

Chapter 1

Introduction

1.1 What is GAMETES?

Genetic Architecture Model Emulator for Testing and Evaluating Software (GAMETES) is a fast direct algorithm for the generation of complex genetic models for simulation studies. In particular, GAMETES is designed to generate epistatic models which we refer to as pure and strict. Purely and strictly epistatic models constitute the worst-case in terms of detecting disease associations, since such associations may only be observed if all n-loci are included in the disease model. This makes them an attractive gold standard for simulation studies considering complex multi-locus effects. The user friendly GAMETES software affords users the ability to rapidly and precisely generate epistatic multi-locus models, as well as the option to generate simulated datasets based on these models.

1.2 Algorithm Overview

This algorithm provides a direct approach for the simulation of biallelic n-locus epistatic models which may be used in conjunction with any sample generation strategy. Each n-locus model is generated deterministically, based on a set of random parameters, a randomly selected direction, and specified values of heritability, minor allele frequencies, and population disease prevalence. For valid combinations of these model constraints, GAMETES attempts to generate a population of model architectures. We use the term *architecture* to reference the unique composition of a model (i.e. the penetrance values and arrangement of those values across genotypes). This algorithm was designed to maximize the randomness of model generation, given a desired set of genetic constraints.

1.3 Further Reading

- For a complete description of the GAMETES algorithm and an example simulation study, see [5].
- For a complete description of the difficulty metric adopted by GAMETES for model selection, see [4].

- For early applications of the GAMETES model/data simulation strategy to a simulation study, see [6, 3].

1.4 Obtaining the Software

GAMETES is available as an open-source (GPL) software package. It is a cross-platform program written entirely in Java. It is freely available for download from http://sourceforge.net/projects/gametes/files/. You may also contact Dr. Jason Moore for a copy of the software or source code if you experience difficulties downloading it from the web site.

1.5 Minimum System Requirements

- Java Runtime Environment, version 5.0 or higher (http://www.java.com/).
- 1 GHz processor
- 256 MB Ram
- 800x600 screen resolution

1.6 Starting the Program

After downloading the file, there will be a file called `GAMETES.jar`. Under most operating systems, simply double-clicking this file will be sufficient to start the program. However, there are reasons a user may wish to start the program from the command line. To do so, open a command shell and navigate to the directory containing `GAMETES.jar`. Issue the command:

```
java -jar GAMETES.jar
```

To open the command line help for GAMETES, give the command:

```
java -jar GAMETES.jar -h
```

Running GAMETES from the command line requires arguments which are discussed in section 2.7. The option to run GAMETES from the command line with no graphical user interface (GUI) facilitates the generation of an extensive model/dataset archive.

Chapter 2

Using GAMETES

2.1 GAMETES GUI Overview

When you open the GAMETES GUI, you will see the window given in Figure 2.1. The GUI is divided into a top half, dedicated to model generation, and a bottom half dedicated to dataset generation. The GAMETES GUI may be used to (1) generate new models, (2) generate datasets from an existing model, or (3) both tasks. As seen in Figure 2.1, when you first start GAMETES, the `Model Construction` box is empty (i.e. there are no model files opened). Whether your task is model or dataset generation, begin by opening a model in the `Model Construction` box. This is accomplished by either clicking the `Create Model` button, or the `Load Model` button at the top of the GUI. The `Create Model` button will open a secondary window for generating a model as discussed in section 2.2. Once a new model has been generated it will automatically open in the `Model Construction` box (see Figure 2.2). The `Load Model` button brings up a file browser, which allows you to navigate to and select your previously saved model file. The `Delete Model` button clears the `Model Construction` box. We discuss dataset generation in section 2.3.

2.2 Model Generation

When the `Create Model` button is selected, the window shown in Figure 2.3 will appear. This window allows the user to specify characteristics of the genetic model they wish to generate. Specifically the user must specify the desired (1) number of attributes, (2) heritability, and (3) minor allele frequencies. Additionally, the user has the option to specify population prevalence. To modify one of the default model constraints, click on the respective box and type in a new value. Double clicking one of these boxs will highlight the contents of the box and allow the user to directly replace the existing value.

2.2.1 Number of attributes

`Number of attributes` refers to the number of SNPs which are to be included in the model. After changing the number of attributes, click on any other white

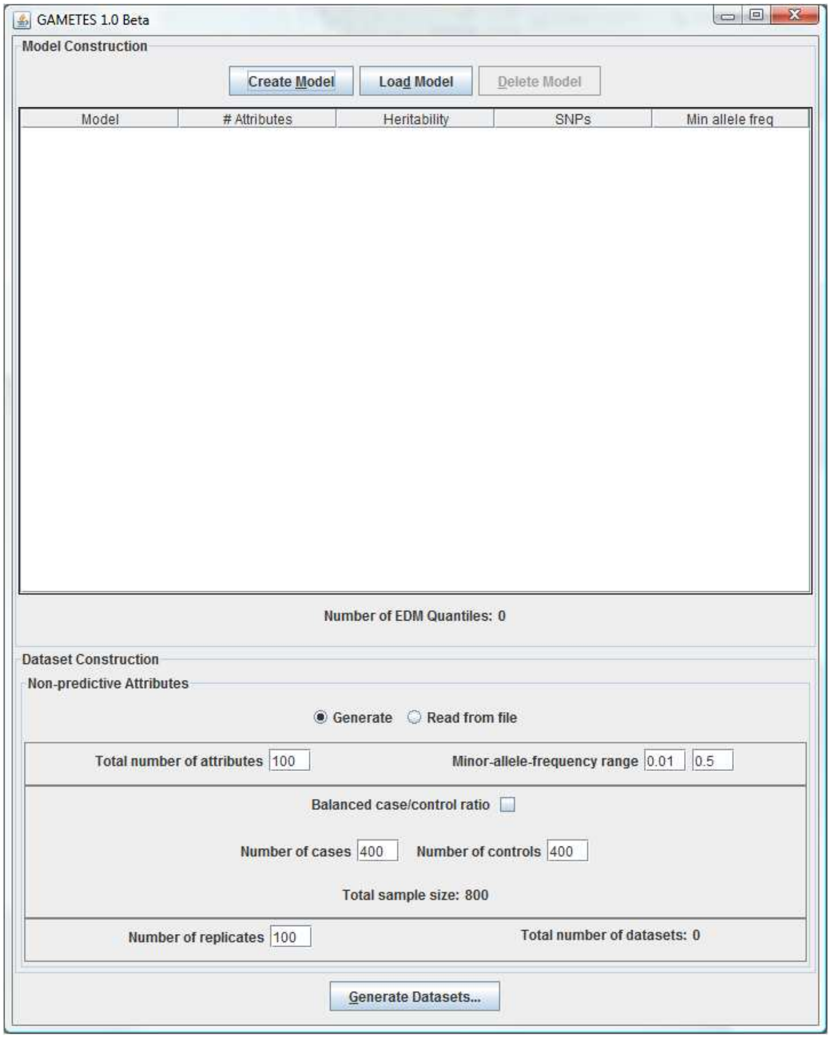

Figure 2.1: GAMETES GUI screenshot

space in the window for the change to take effect. You will see additional rows appear in the model window as you increase this value.

2.2.2 Heritability

`Heritability`, or the (broad-sense) heritability of a genetic model is the proportion of observable differences between individuals that is due to genetic differences. Keep in mind that as you select higher values of heritability it becomes less likely that GAMETES will be able to randomly generate such models since GAMETES has the ability to scale a model down to the desired heritability, but not up. See [5] for more details.

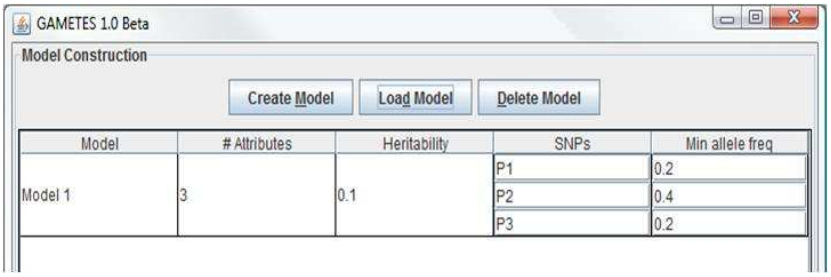

Figure 2.2: Model opened in the model construction box.

2.2.3 Population Prevalence

Population prevalence (K) is the proportion of individuals in a population that have the disease of interest. Note that GAMETES gives the user the option to leave K unspecified, in which case the K of the models that are generated will vary. To specify K, select the check-box to the left of `Prevalence` and then specify a value of K between 0 and 1. If this box is left un-checked, K will be allowed to vary. Previous analysis of simulated models suggests that K has a negligible impact on model difficulty.

2.2.4 Minor Allele Frequency

The minor allele frequency (MAF) is the frequency at which the less common allele occurs in a given population. Once the user has specified number of attributes, adjust the MAF for every SNP in the model to the desired value between 0 and 0.5.

2.2.5 Other Parameters

While GAMETES gives a default name for each SNP in the model (e.g. P1, which stands for Predictive SNP 1) you may modify each name, again by clicking on the respective cell and typing in a new name. Two additional parameters (i.e. `Quantile Count` and `Quantile Population Size`) specify the number of models which will be generated and selected to be saved by GAMETES. If you wish to generate a single random model (with the specified model constraints) set both of these parameters to 1. Each model that is successfully generated will satisfy the four model constraints given, but will possess a unique random architecture. `Quantile Count` refers to the number of random model architectures you wish to save to the model output file (which may subsequently used to generate simulated datasets). `Quantile Population Size` refers to the number of random model architectures you want GAMETES to generate for the given model constraints. If you want GAMETES to save all model architectures it generates, set `Quantile Count` equal to `Quantile Population Size`. Setting `Quantile Count` to a lower value than `Quantile Population Size` will direct GAMETES to choose a subset of the random models to be saved. This selection process is based on a model difficulty metric discussed in section 2.4. In short, N random models (where N = `Quantile Population Size`) are ordered by their difficulty and X models are reported/saved (where X = `Quantile`

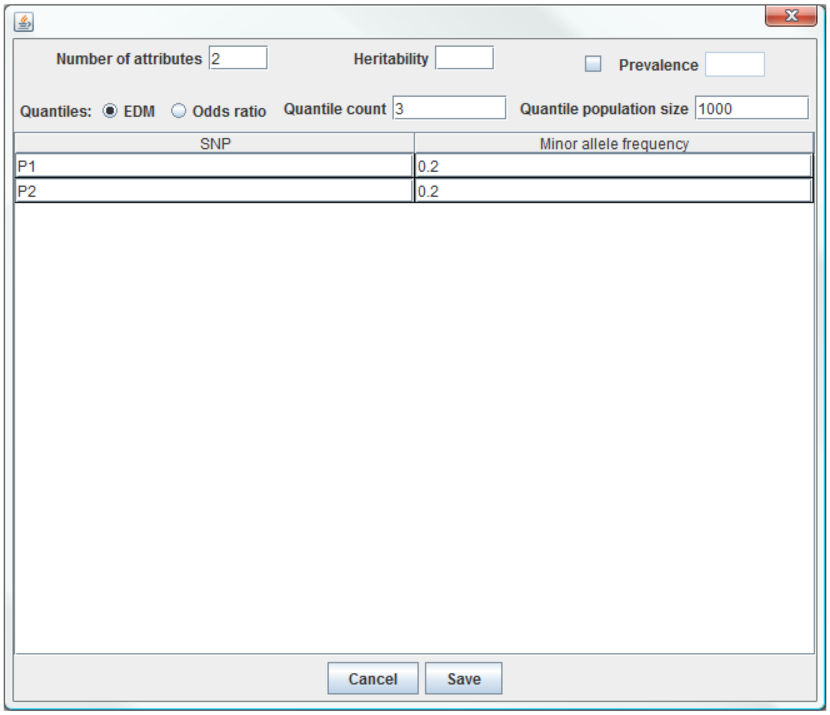

Figure 2.3: Model generation window.

Count). Quantile Count may not be larger than Quantile Population Size. If Quantile Count = 1, the model with the median "difficulty" is reported. If Quantile Count = 2, the models with the maximum and minimum "difficulties" are reported. If Quantile Count = 3, the maximum, minimum, and median models are reported. Larger values of Quantile Count select models at evenly spaced intervals. You may also specify one of two difficulty metric options; EDM or COR to be used in model selection. See Section 2.4 for more on these metrics.

2.2.6 Saving Models

Once all parameters have been specified, click Save. This will bring up a file browser, which allows you to select the name and destination for your model file. Model files are saved as YourName_models.txt. In addition to the models file, a secondary txt file will be saved that gives the model difficulty scores for all N models generated for the given combination of model constraints. These files are saved as YourName_EDMscores.txt or YourName_CORscores.txt depending on the difficulty metric you select. When searching for models, a progress bar will appear which indicates the number proportion of search attempts GAMETES has made. This does not necessarily reflect the number of models it has successfully found during the course of running.

2.3 Dataset Generation

Once a model has been opened in the `Model Construction` box, you may generate simulated datasets derived from that model. Notice that the `Number of Quantiles` for the loaded model is displayed at the bottom of the `Model Construction` box. Datasets simulated using GAMETES have two types of attributes/SNPs, (1) predictive attributes, and (2) non-predictive attributes. Predictive attributes are those specified in the genetic model. Non-predictive attributes are all other attributes which have no specified association with affection status (i.e. case or control). The first step is to decide how to include non-predictive attributes. Select the radio button, `Generate` to randomly generate genotypes for all non-predictive attributes. Select the radio button `Read from file` to use an existing real or simulated SNP dataset as the non-predictive attributes.

2.3.1 Randomly Generate Non-Predictive Attributes

If `Generate` is selected the GAMETES window will appear as in Figure 2.1. If `Generate` is selected then you must also specify the `Total number of attributes`. This is the total number of SNPs which will appear in each simulated dataset. This number includes all predictive attributes included in the model. E.g. If you have a 3-locus model, and you specify 100 total attributes, 97 will be non-predictive. Additionally, select the `Minor-allele-frequency range`. The MAF of each non-predictive attribute is randomly selected from within this range with uniform probability. Next if an equal number of cases and controls is desired, check the box for `Balanced case/control ratio`. This will restrict the number of controls, such that it is the same as the number of cases. Adjust `Number of cases` and `Number of controls` by double-clicking the respective box and entering the desired value. If `Balanced case/control ratio` is checked, a change in `Number of cases` will appear in `Number of controls` when you click in the white space of a separate box. Once you have done so, the `Total sample size` will update it self to reflect the correct total. Finally, specify the `Number of replicates`. This is the number of randomly seeded simulated datasets which will be generated for each of the genetic models. `Total number of datasets` indicates the number of datsets which will be generated when the user clicks `Generate Datasets...` at the bottom of the window. If the user has selected 3 model quantiles, and 100 replicates, a total of 300 datasets will be generated (100 for each of three models with different architectures, but the same set of model constraints). Next, click `Generate Datasets...`. This will bring up a file browser, which allows you to select the name and destination for folders which will contain your saved datasets (one folder for each quantile). Finally, click `Save` and a progress bar will appear as datasets are generated and saved.

2.3.2 Load Non-Predictive Attributes

If you select `Read from file`, the options in the lower half of the GAMETES window change (see Figure 2.4). Selecting this option allows a user to load an existing file (such as a real SNP dataset) to be used as the non-predictive attributes. Click `Load SNP file` to bring up a file browser, which allows you

to navigate to and select an existing dataset. The current implementation of GAMETES requires the following format for loaded datasets:

- Files should be tab-delimited text files.
- The first row of the file should contain header labels for all attributes/SNPs.
- Rows should denote subjects, and columns should denote attributes/SNPs.
- The three biallelic SNP genotypes should be encoded as 0,1,or 2. There should be no other attribute values or missing data.
- The file should include attribute data only (no class label or status for any subject).

Once loaded, the `Number of attributes` and `Total number of instances` will be automatically determined, based on the dataset that has been loaded. `Number of attributes` will equal the number of attributes in the loaded dataset only. The number of attributes in the datasets that will be generated is `Number of attributes` plus the number of attributes in the loaded genetic model. `Total number of instances` will be equal to the number of instances in the loaded dataset. Click `Generate Datasets...` to generate datasets as described above.

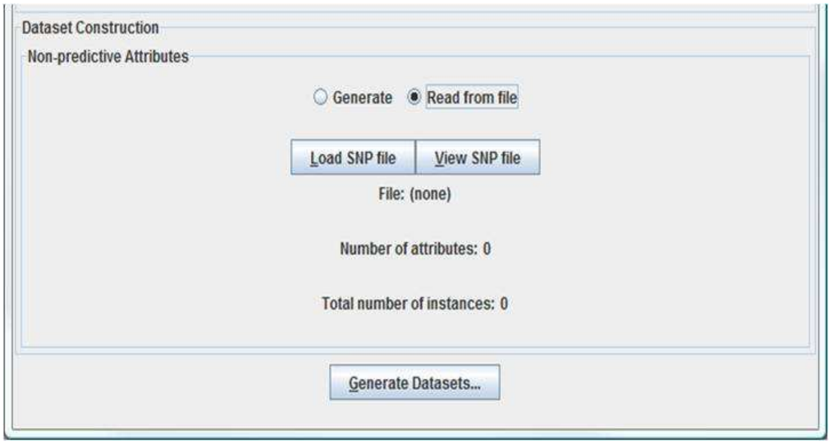

Figure 2.4: Read from file options

2.4 EDM and COR

A more recent feature to have been added to GAMETES is the ability to select a subset of representative models from a population of models with random architectures. In developing and testing GAMETES, we observed that despite keeping all previously mentioned model constraints constant, an algorithm's detection proportion could vary greatly. Detection proportion refers to the proportion of datasets within which the correct underlying model was identified. One would observe higher proportions for detecting an "easier" model, than a

more "difficult" model. While some variation can be explained by the probabilistic translation of models into randomly seeded datasets, the rest can logically be attributed to subtle differences in model architecture. GAMETES has two difficulty metrics implemented as options for model selection. These include a customized odds ratio (COR), informally utilized in [2] and [1], as well as our Ease of Detection Measure (EDM), formally introduced and evaluated in [4]. Both metrics are calculated directly from the genetic model, and were found to demonstrate a strong, significant correlation with a given model's detectability. Both metrics offer a viable method for model selection with no significant difference between them.

2.5 Model Output Files

Text files in which generated genetic models are saved provide the following information for each model selected for reporting by GAMETES: attribute names, MAFs, K, heritability, and either the COR or EDM score for the model (depending on what the user selected as the difficulty metric). Additionally the penetrance values for every genotype combination in the model are given as a penetrance function. These penetrance functions can become difficult for a user to easily interpret as the number of loci (n) is greater than 3. If n is 2, the genotypes of the first SNP are the rows of the penetrance function, while the genotypes of the second SNP are the columns. If n is 3, these positions shift. The genotypes of first SNP are represented by the three 2-locus penetrance functions, the genotypes of the second SNP are the rows, and the genotypes of the third SNP are the columns. If n is 4, the genotypes of first SNP are now represented by the three, 3-locus penetrance functions. This pattern continues as n continues to increase, where the last of n SNPs is always represented by the columns. See Table 2.1 below for an example of a 2-locus penetrance function, where the function includes 9, or 3^n, penetrance values. Notice here how the rows represent genotypes of SNP 1, while columns represent genotypes of SNP 2.

Table 2.1: A 2-locus purely epistatic penetrance function.

		SNP 2			Marginal
	Genotype	BB(.25)	Bb (.5)	bb(.25)	Penetrance
SNP 1	AA(.36)	.266	.764	.664	.614
	Aa (.48)	.928	.398	.733	.614
	aa(.16)	.456	.927	.147	.614
	Marginal Penetrance	.614	.614	.614	K = .614

2.6 Limits of GAMETES

In [5] we discuss specific limits of the GAMETES software. In particular users should be aware that GAMETES's ability to generate genetic models is limited by what constraint combinations are mathematically possible, as well as which ones have a reasonable probability of being generated by chance. We

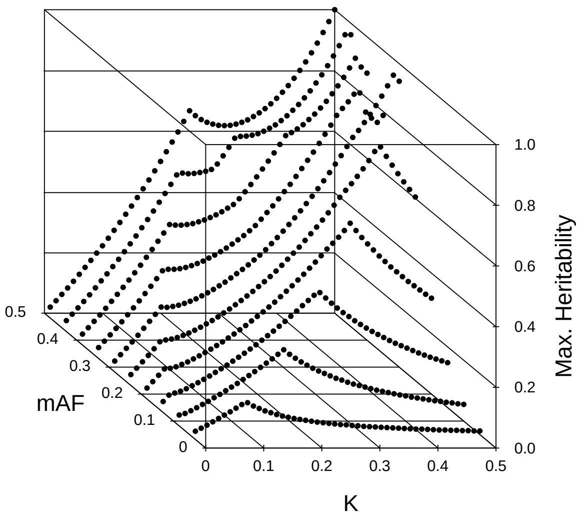

Figure 2.5: Plot of our maximum heritability estimates for pure, strict, 2-locus models.

illustrate the limits of 2-locus model constraint combinations in Figure 2.5 published in [5]. Presently if you wish to know if GAMETES can generate models with a particular set of model constraints, the best strategy is trial and error. By default, the GAMETES GUI is alotted a `Number of Attempts` of 100,000 to find the desired number of models for `Quantile Population Size` requests up to 1000. If the user requests population sizes larger than 1000, the maximum `Number of Attempts` becomes 100X the requested `Quantile Population Size`. Alternatively, `Number of Attempts` may be precisely specified when running GAMETES from the command-line. If GAMETES fails to find the number of models specified in `Quantile Population Size`, but that number is larger than Quantile Count (the number of models the user wanted specified in the model file), a warning message will appear notifying the user of the number of models it was able to find. In this case a model file will still be generated, but GAMETES will select the number of models specified in Quantile Count, out of the population of models it was able to find. If GAMETES finds fewer models than Quantile Count, the following error message will appear: "Unable to generate desired number of table quantiles". In this situation GAMETES will not output a model file.

Generally speaking if GAMETES was unable to find models with the desired constraints, try using a larger `Number of Attempts`, especially when looking for models with higher heritabilities (e.g. > 0.4) and higher n. If this still does not work, either the combination requested is not mathematically possible, or the odds of randomly generating it are highly improbable.

2.7 Command-Line Operation

GAMETES may also be run from the command line. If your goal is to generate an archive of genetic models and simulated datasets, than it would be advantageous to code a wrapper script calling GAMETES from the command line. To obtain a list of commands for command line operation, type `java -jar GAMETES.jar -h`. Like the GUI, running GAMETES from the command line affords users the ability to independently generate genetic models and then separately generate simulated datasets.

As an example, the following command would direct GAMETES to generate a 2-locus model with heritability (0.2), population prevalence (0.1), one SNP with MAF (0.3), and the second with MAF (0.2).

```
java -jar GAMETES.jar -M " -h 0.2 -p 0.1 -a 0.3 -a 0.2" -q 3 -p
        1000 -t 100000 -o mySimulatedModel.txt
```

GAMETES would output (3) model architectures to a file named "mySimulatedModel.txt" in the working directory. These models would be selected from a population of 1000 models generated by GAMETES with the above model constraints. Since GAMETES does not generate a successful model every attempt, in this example we have limited GAMETES to 100,000 model generation attempts, at which point it will stop trying to reach its goal of 1000 models, and select the (3) specified model architectures, assuming it has found at least three in its search.

As an example of dataset generation, the following command would direct GAMETES to generate 100 replicate datasets from each model architecture found in "mySimulatedModel.txt".

```
java -jar GAMETES.jar -i mySimulatedModel.txt -D " -n 0.01 -x 0.5
     -a 100 -s 500 -w 500 -r 100 -o mySimulatedDataset"
```

For each of these datasets, non-predictive SNPs will have a minimum MAF of 0.01, and a maximum MAF of 0.5. Each will also have a total of 100 attributes (predictive and non-predictive combined), along with 500 cases and 500 controls.

Chapter 3

Intended GAMETES Expansions

The following sections describe expansions of the GAMETES software intended to be made available in future versions.

3.1 Heterogeneous Models

In order to generate simulated datasets displaying genetic heterogeneity, we plan to add the ability to load multiple models into the `Model Construction` box, and allow the user to specify the proportion of the dataset which will be generated from each model. This will simulate genetic heterogeneity by independently generating samples from respective models.

3.2 Impure and Nested Epistasis

While pure, strict epistasis makes a logical gold standard for complex multi-locus interaction models, users may want to generate models that don't meet these strict specifications. The GAMETES algorithm may be easily expanded to generate impure, nested epistatic models as well. *Impure* epistasis implies that one or more of the interacting loci have a main effect contributing to disease status. *Nested* refers to epistasis in which at least one proper subset of the loci also interact epistatically.

3.3 Custom Genetic Models

In order to expand the utility and flexibility of GAMETES we intend to add the option for users to uniquely specify their own 1 to 3-locus models, by manually entering the 3^n penetrance values, and n MAFs of the given model. This option will automatically calculate the heritability, population prevalence and marginal penetrance values of the resulting model. This will allow users to explore the characteristics of smaller genetic models and directly generate simulated datasets from these models specified by the user. Users may also enter models with classically recognizable genetic patterns.

Bibliography

[1] T. Edwards, K. Lewis, T. Digna, R. Dudek, and M. Ritchie. Exploring the performance of multifactor dimensionality reduction in large scale SNP studies and in the presence of genetic heterogeneity among epistatic disease models. *Hum. Hered*, 67:183–192, 2009.

[2] A.A. Motsinger-Reif, D.M. Reif, T.J. Fanelli, and M.D. Ritchie. A comparison of analytical methods for genetic association studies. *Genetic epidemiology*, 32(8):767–778, 2008.

[3] R. Urbanowicz and J. Moore. The application of pittsburgh-style learning classifier systems to address genetic heterogeneity and epistasis in association studies. *Parallel Problem Solving from Nature–PPSN XI*, pages 404–413, 2011.

[4] R.J. Urbanowicz, J. Kiralis, J. Fisher, and J.H. Moore. Predicting the Difficulty of Pure, Strict, Epistatic Models: A Metric for Simulated Model Selection. *BMC Bioinformatics, Submitted*, 2011.

[5] R.J. Urbanowicz, J. Kiralis, N. Sinnott-Armstrong, T. Heberling, J. Fisher, and J.H. Moore. GAMETES: A Fast, Direct Algorithm for Generating Pure, Strict, Epistatic Models with Random Architectures. *BMC Bioinformatics, Submitted*, 2011.

[6] R.J. Urbanowicz and J.H. Moore. The application of michigan-style learning classifier systems to address genetic heterogeneity and epistasis in association studies. In *Proceedings of the 12th annual conference on Genetic and evolutionary computation*, pages 195–202. ACM, 2010.
